# Aryloxide‐Facilitated Catalyst Turnover in Enantioselective α,β‐Unsaturated Acyl Ammonium Catalysis

**DOI:** 10.1002/anie.201706402

**Published:** 2017-08-25

**Authors:** Anastassia Matviitsuk, Mark D. Greenhalgh, Diego‐Javier Barrios Antúnez, Alexandra M. Z. Slawin, Andrew D. Smith

**Affiliations:** ^1^ EaStCHEM School of Chemistry University of St Andrews North Haugh St Andrews Fife KY16 9ST UK

**Keywords:** aryloxides, isothiourea, kinetic analysis, Lewis base catalysis, α,β-unsaturated ammonium compounds

## Abstract

A new general concept for α,β‐unsaturated acyl ammonium catalysis is reported that uses p‐nitrophenoxide release from an α,β‐unsaturated p‐nitrophenyl ester substrate to facilitate catalyst turnover. This method was used for the enantioselective isothiourea‐catalyzed Michael addition of nitroalkanes to α,β‐unsaturated p‐nitrophenyl esters in generally good yield and with excellent enantioselectivity (27 examples, up to 79 % yield, 99:1 er). Mechanistic studies identified rapid and reversible catalyst acylation by the α,β‐unsaturated p‐nitrophenyl ester, and a recently reported variable‐time normalization kinetic analysis method was used to delineate the complex reaction kinetics.

Lewis base organocatalysis is a widely studied field due to the diverse range of molecular frameworks that can be produced with high levels of regio‐, chemo‐ and stereocontrol.^1^ At the carboxylic acid oxidation level a variety of ammonium intermediates with differing reactivity can be accessed from readily available substrates using tertiary amine Lewis bases (Scheme 1 a). Acyl ammonium and ammonium enolate intermediates have been extensively studied and applied in enantioselective acyl transfer processes and formal cycloadditions, respectively.^2^, ^3^ A less studied but equally powerful reactivity mode is that of α,β‐unsaturated acyl ammonium intermediates.^4^ These species contain electrophilic centres at the C1 and C3 positions, and a latent nucleophilic centre at C2, providing new opportunities for reaction design to target previously inaccessible product architectures.^5^

**Scheme 1:**
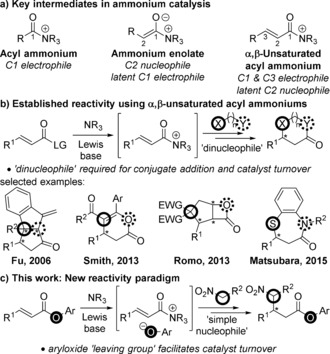
Nomenclature, reactivity and applications of ammonium intermediates in catalysis.

Seminal work by Fu first demonstrated the feasibility of this concept in a formal [3+2] cycloaddition using α,β‐unsaturated acyl fluorides as the α,β‐unsaturated acyl ammonium precursor (Scheme 1 b).^6^ Recent studies from ourselves, Romo, and Matsubara, has built on this precedent to achieve highly enantioselective Michael addition‐annulation, formal cycloaddition and complex cascade methodologies.^7^ These examples used α,β‐unsaturated acid anhydrides or halides as the α,β‐unsaturated acyl ammonium precursors. In addition, these methodologies require the reactive partner to contain two distinct nucleophilic functionalities to 1) undergo conjugate addition to the α,β‐unsaturated acyl ammonium intermediate, and 2) enable turnover of the Lewis base catalyst (Scheme 1 b). This requirement inherently limits α,β‐unsaturated acyl ammonium catalysis and must be overcome to allow more diverse processes. In addition only preliminary experimental mechanistic work has been undertaken, with no kinetic analysis reported to date.^8^

Here we report the development of a new general concept for α,β‐unsaturated acyl ammonium catalysis. Catalyst turnover is not facilitated by the nucleophilic reaction partner, but by an aryloxide counterion released in situ during the reaction by using an α,β‐unsaturated aryl ester as the α,β‐unsaturated acyl ammonium precursor (Scheme 1 c).^9^, ^10^, ^11^ This allows the use of simple nucleophiles as reaction partners, providing enhanced potential for further advancement of the field. Mechanistic work including kinetic analysis, catalyst labeling and crossover studies are also reported to deliver a fundamental understanding of this process.

As initial proof of concept, the Michael addition of nitroalkanes to α,β‐unsaturated aryl esters using a Lewis basic isothiourea catalyst was investigated.^12^ Although the organocatalytic enantioselective Michael addition of nitroalkanes to enones or enals is well precedented,^13^ Lewis base catalysis of this process has not been demonstrated at the carboxylic acid oxidation level.

Initial investigations focused on the reaction of a range of α,β‐unsaturated aryl esters **1**–**4**, bearing different aryl groups, with excess nitromethane using HyperBTM **5** as catalyst (Table 1, entries 1–4). The Michael addition products **6**–**9** were formed in each case in moderate to excellent yield (48–81 %) but with uniformly high enantioselectivity (up to 96:4 er) and with complete regioselectivity.^14^ The highest yields were obtained using *p*‐nitrophenyl (PNP) and 3,5‐bis(trifluoromethyl)phenyl esters **1** and **4**, with PNP ester **1** chosen for further studies due to the higher enantioselectivity obtained. Mixed solvent systems proved ineffective, with lower yields obtained in the presence of both THF and MeCN (entries 5 and 6). The addition of a base (2,6‐lutidine) did not prove beneficial (entry 7),^15^ whilst heating the reaction at 70 °C resulted in complete decomposition (entry 8). Alternative isothiourea catalysts did not provide improved results, and lower catalyst loadings resulted in incomplete conversion, which complicated product isolation.^16^

**Table 1:** Reaction optimization. 
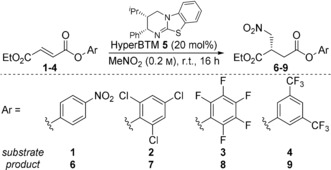

Entry	Subst.	Solvent	Additive (equiv)	Yield [%]^[a]^	er^[b]^
1	**1**	*neat*	–	81 (55)	96:4
2	**2**	*neat*	–	54 (41)	94:6
3	**3**	*neat*	–	48 (33)	95:5
4	**4**	*neat*	–	78 (45)	93:7
5	**1**	MeNO_2_:THF (1:1)	–	50	ND^[c]^
6	**1**	MeNO_2_:MeCN (1:1)	–	43	ND^[c]^
7	**1**	*neat*	2,6‐lutidine (0.2)	63	ND^[c]^
8^[d]^	**1**	*neat*	–	0	–

[a] Determined by ^1^H NMR spectroscopic analysis using 1,4‐dinitrobenzene as internal standard (isolated yields given in parentheses). [b] Determined by chiral HPLC analysis. [c] ND=not determined. [d] Reaction performed at 70 °C.

The scope and limitations of the method was then investigated. Given the moderate isolated yields of PNP ester products, the addition of a suitable nucleophile at the end of the reaction was used to give a range of readily isolable functionalized products (Table 2). The use of primary and secondary amines gave secondary and tertiary amides **10**–**14** in good yield, whilst addition of methanol gave methyl ester **15**. All amide and ester products were obtained with high enantioselectivity indicating no significant loss in enantiopurity during the derivatization process. The scope of β‐substituted α,β‐unsaturated aryl esters amenable to the process was then investigated. Methyl‐, isopropyl‐ and benzyl esters gave the addition products **16**–**18** in good yield and with excellent enantioselectivity. The incorporation of amides at the β‐position was also well tolerated, giving unsymmetrical succinamide derivatives **19** and **20** in equally high yield and levels of enantiocontrol. The absolute configuration of **19** was confirmed by single crystal X‐ray analysis, with all other examples assigned by analogy.^17^ Limitations of this methodology include incompatibility of substrates such as γ‐keto ester derivative **22**, which gave a complex mixture of products, and cinnamic acid derivative **23**, which was completely unreactive. A derivative bearing β‐alkyl substitution however gave product **21** with excellent enantiocontrol, albeit in low yield. The synthesis of a quaternary stereogenic carbon centre was also attempted, however application of β,β‐disubstituted derivative **24** failed to give the desired Michael addition product.

**Table 2:** Reaction scope: Variation of α,β‐unsaturated *p*‐nitrophenyl ester and nucleophilic quench.^[a]^

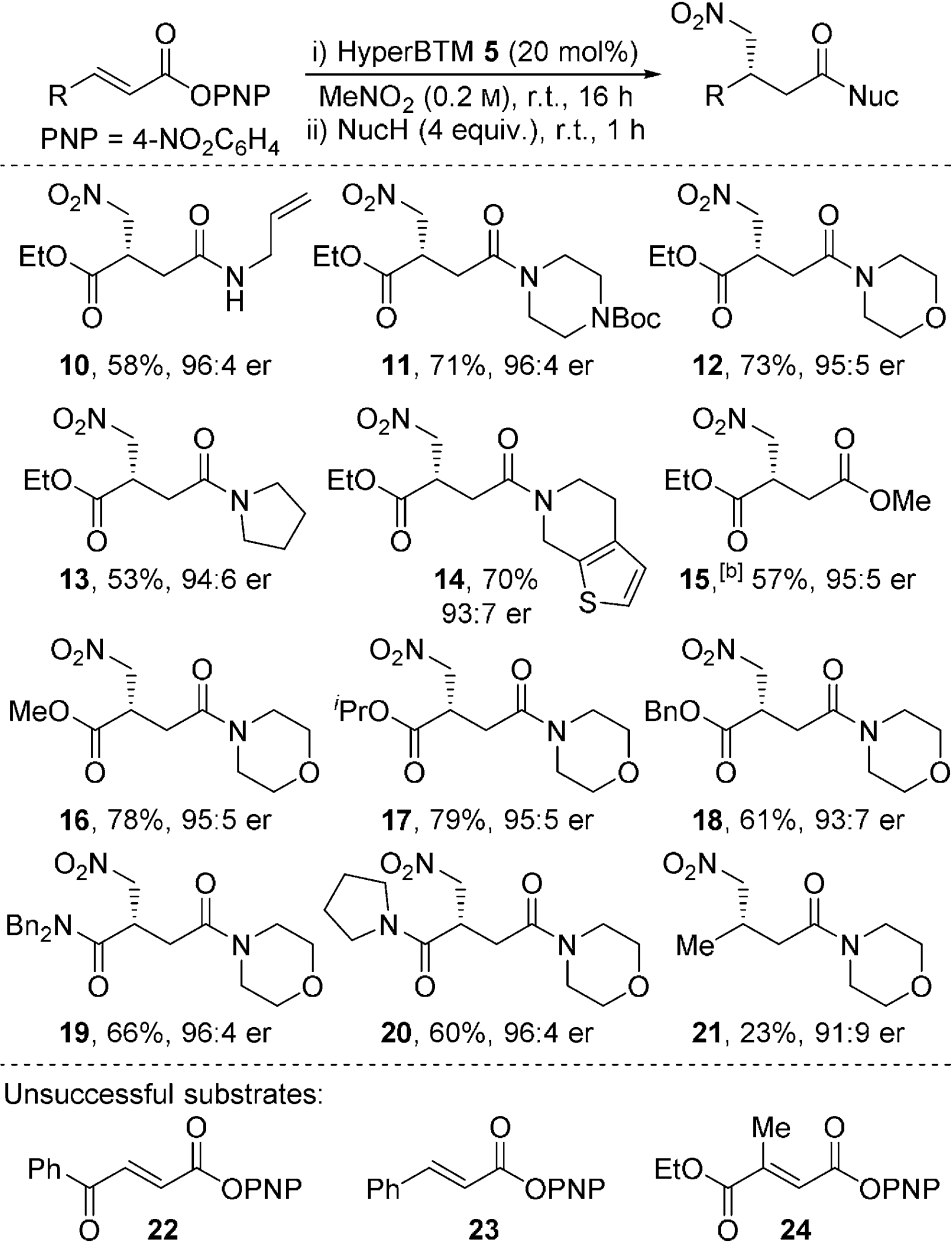

[a] Isolated yields given; er determined by chiral HPLC analysis. [b] Excess MeOH and DMAP (20 mol %) used in step ii).

The effect of olefin configuration was investigated using maleate PNP ester derivative **25** (Scheme 2). Interestingly, the Michael addition product **12** was obtained in the same enantiomeric form (93:7 er) as when using the isomeric fumarate PNP ester **1** (95:5 er). Monitoring reaction progress by ^1^H NMR spectroscopy revealed rapid isomerization of maleate **25** to fumarate PNP ester **1** on a faster timescale than formation of product, with control reactions in [D_6_]DMSO indicating reversible aryloxide conjugate addition as a possible mechanism for this isomerization process.^16^, ^18^

**Scheme 2:**
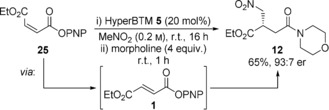
Michael addition using maleate derivative **25**.

Attention was next turned to the use of alternative nitroalkanes and subsequent derivatization of the products. Nitroethane and nitropropane were suitable nucleophiles giving addition products **26** and **27** in good yield. Although only minimal diastereocontrol was observed, both diastereoisomers were obtained with excellent enantioselectivity (99:1 er, Table 3). Pleasingly, the use of 2‐nitropropane and nitrocyclopentane was also successful, giving amide and ester products **28**–**31** in moderate yield but with excellent enantiocontrol.

**Table 3:** Reaction scope: Nitroalkane variation.^[a]^

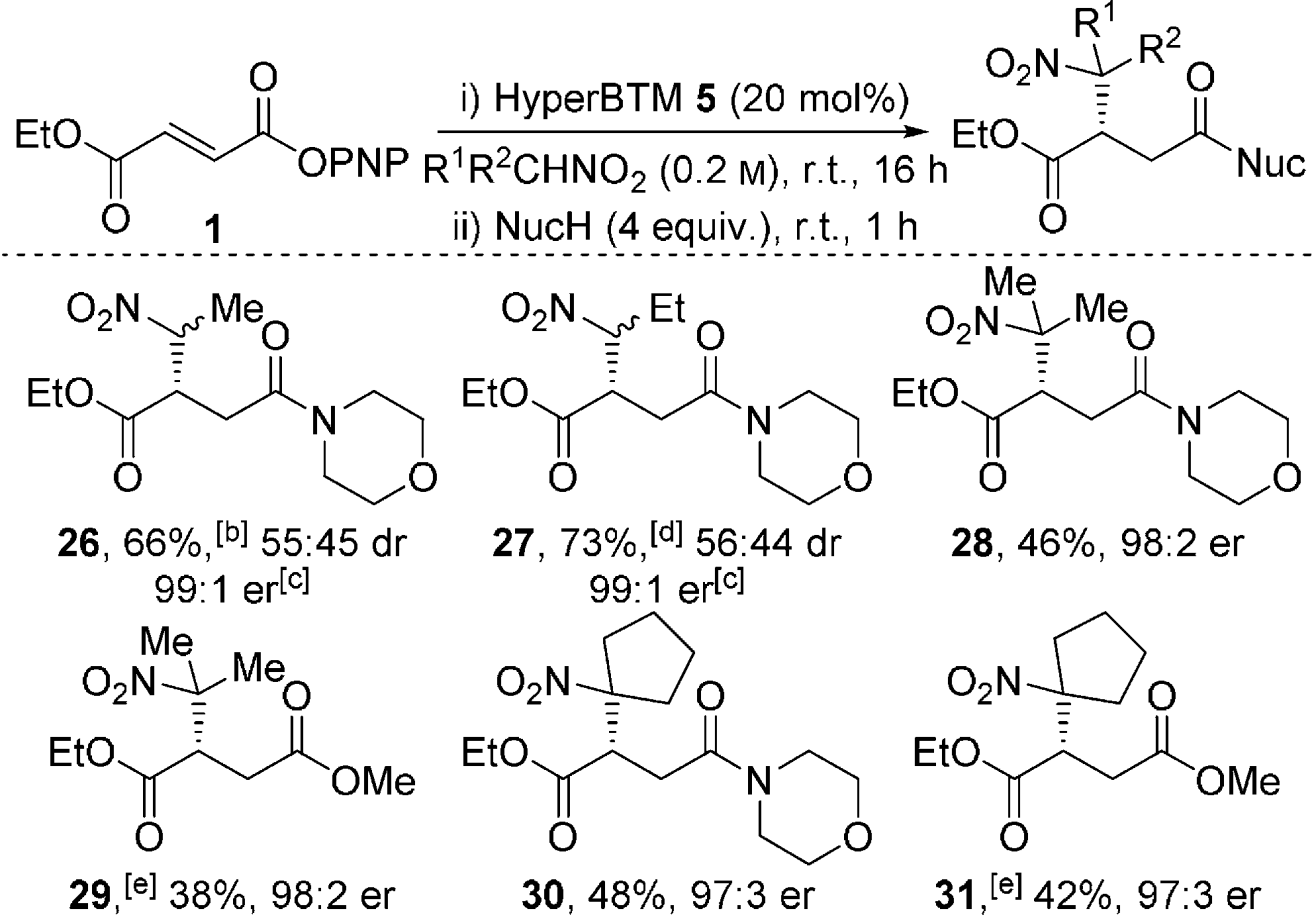

[a] Isolated yields given; dr of crude product determined by ^1^H NMR spectroscopic analysis (relative configurations not confirmed); er determined by chiral HPLC analysis. [b] Isolated as a mixture of diastereoisomers. [c] er of both diastereoisomers. [d] Diastereoisomers separated by column chromatography [41 % (major); 32 % (minor)]. [e] Excess MeOH and DMAP (20 mol %) used in step ii).

Reduction of γ‐nitro methyl esters **15**, **29** and **31** and subsequent cyclization was achieved with no loss in enantiopurity to give pyrrolidinone derivatives **32**–**34** in excellent yield and highly enantioenriched form (Table 4).^19^ The biological importance of pyrrolidinones, and γ‐aminobutryric acid (GABA) derivatives in general, is well precedented.^20^

**Table 4:** Product derivatization: Synthesis of enantioenriched pyrrolidinones.^[a]^

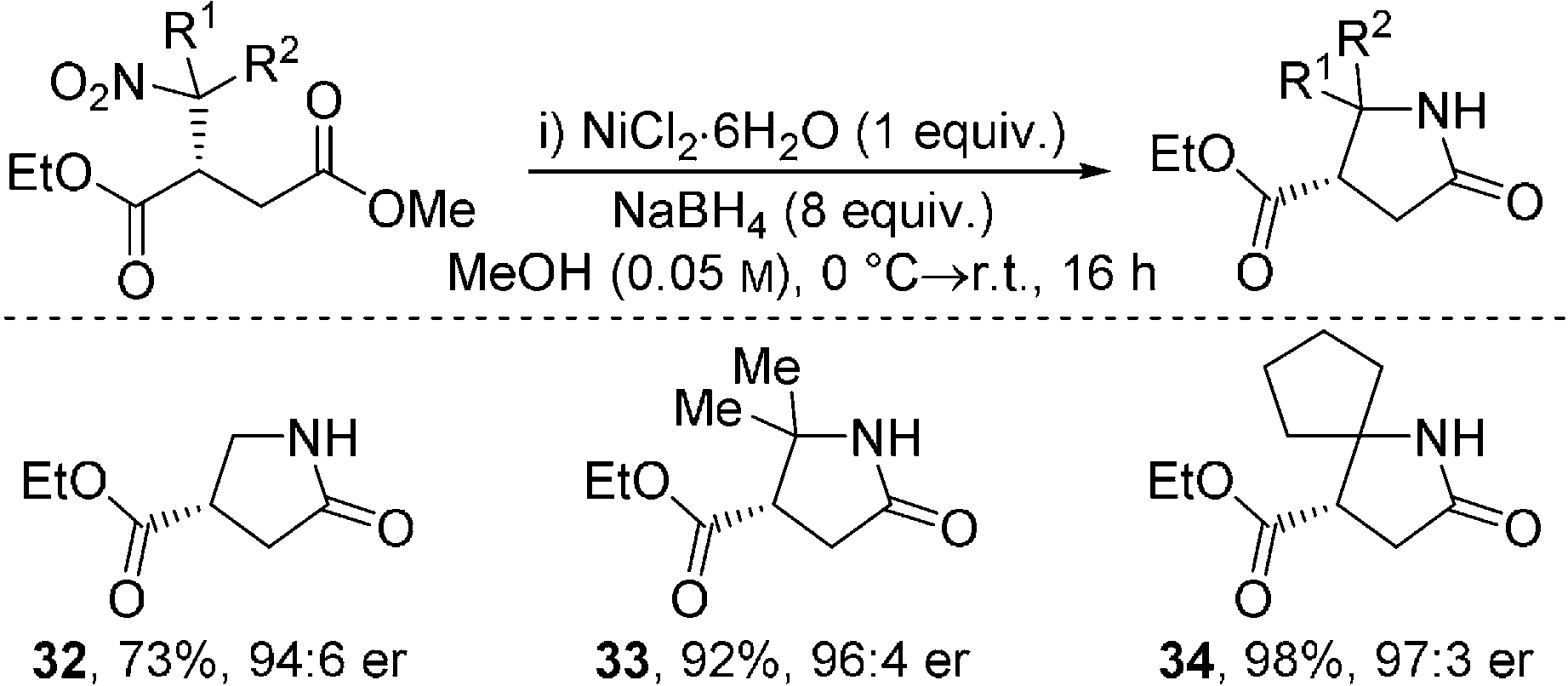

To provide greater insight into this methodology, the reaction mechanism and kinetics were investigated to identify reaction intermediates and determine the reaction order with respect to each component. Quantitative reaction monitoring was achieved by in situ ^19^F{^1^H} NMR spectroscopy using ^19^F‐labeled PNP ester **35** and (2*R*,3*S*)‐8F‐HyperBTM **36** in MeNO_2_ using PhF as internal standard and a C_6_D_6_‐filled capillary reference (Figure 1 a,b). Attempts to interrogate the kinetic data revealed a substantial reduction in reaction rate over the course of the reaction, suggesting deactivation of the catalyst. During the reaction, the ^19^F chemical shift (*δ*_F_) of (2*R*,3*S*)‐8F‐HyperBTM **36** underwent a significant downfield drift (*δ*_F_=−122.68 → ≈−119.6 ppm), indicative of an equilibrating mixture of protonated and freebase isothiourea. Using an independently synthesized sample of **36**⋅HCl as a reference (*δ*_F_=−116.72 ppm), the proportion of freebase isothiourea **36** in the reaction was calculated as a function of its chemical shift (Figure 1 b, **×**).^16^, ^21^ Low concentrations (≤0.4 mm) of proposed acyl isothiouronium species **38** (○) and **39** (▵) were also identified by the downfield chemical shift of the isothiouronium fluorine label (*δ*_F_=−111.79 and −111.97 ppm) (Figure 1 a and b, inset).10d Addition of an isolated α,β‐unsaturated acyl isothiouronium **38** (where X=Cl, *δ*_F_=−111.81 ppm)^22^ to a reaction in progress resulted in significant enhancement of both signals, providing support for this assignment. In addition, mixing (2*R*,3*S*)‐8F‐HyperBTM **36** and Michael addition product **37** gave a minor species with *δ*_F_=−111.97 ppm, consistent with nucleophilic addition of **36** to **37** to give the post‐Michael addition acyl isothiouronium **39**.^16^ These studies are consistent with speciation of the isothiourea catalyst between at least four forms, with the dominant, resting state, the freebase isothiourea **36**.

**Figure 1:**
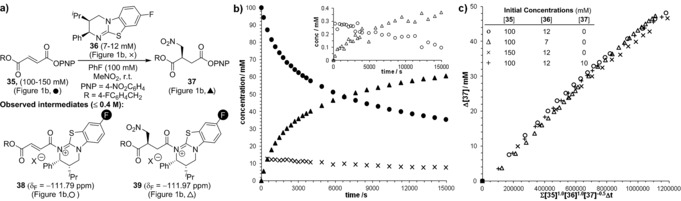
Temporal concentration data and variable time normalization kinetic analysis (VTNKA) for Michael addition of nitromethane to **35** using (2*R*,3*S*)‐8F‐HyperBTM **36**. a) Reaction Scheme. b) Typical reaction profile: initial conditions: **35** (100 mm), **36** (12 mmol) in MeNO_2_ (0.6 mL), PhF (100 mm), C_6_D_6_ capillary reference, RT; Key: •=**35**; ×=freebase **36** (calculated); ▴=**37**; inset: ○=**38**; ▵=**39**. c) VTNKA: *x*‐axis normalization for [**35**], [**36**] and [**37**].

Having established a method for quantifying the temporal concentration of reaction components, determination of the reaction order with respect to each component was sought. The complex catalyst speciation, in addition to slow hydrolysis of starting material over the reaction course, indicated that kinetic analysis may be challenging. However, as the temporal concentrations of each component were easily measured, the innovative variable time normalization graphical analysis method reported recently by Burés was applied.^23^ Kinetic analysis was performed for three reactions with different starting concentrations of α,β‐unsaturated ester **35** and (2*R*,3*S*)‐8F‐HyperBTM **36** (Figure 1 a), with the concentration of MeNO_2_ assumed to remain constant (pseudo‐zero order in MeNO_2_). A plot of concentration of product **37** against a normalized time axis of Σ[**35**]^*α*^[**36**]^*β*^Δ*t* (where *α* and *β* represent the respective reaction orders of each component) allowed graphical interrogation of the kinetic profiles. Systematically varying *α* and *β* provided optimal overlay for *α*=1.0 and *β*=1.0, indicating the reaction is first order in both ester substrate and catalyst.^16^ Despite good overlay, the curvature of the plot suggested an additional reaction variable had been omitted from the analysis. Further studies showed that addition of product **37** (10 mm) at the start of the reaction resulted in rate retardation, consistent with product inhibition.^16^ Incorporation of [**37**]^*γ*^ into the normalized time axis (Σ[**35**]^*α*^[**36**]^*β*^[**37**]^*γ*^Δ*t*) resulted in good overlay and linearity at an arbitrary value of *γ*=−0.5 (Figure 1 c).

A series of crossover reactions was used to investigate the reversibility of the primary catalytic steps (Scheme 3). Treatment of α,β‐unsaturated esters **40** and **41** bearing two distinct PNP ester groups (2‐fluoro and 3‐fluoro) and two distinct β‐substituents (amide and ester) under catalytic conditions was monitored by in situ ^19^F{^1^H} NMR spectroscopy (Scheme 3 a). Rapid equilibration gave a mixture of all four possible α,β‐unsaturated esters **40**–**43** within 5 minutes, with subsequent formation of the four corresponding Michael addition products **44**–**47**.^24^ A second crossover experiment between two Michael addition products, **44** and **45**, bearing distinct PNP ester groups and β‐substituents, also resulted in rapid exchange (Scheme 3 b). These experiments show that the isothiourea undergoes rapid and reversible acylation by both the α,β‐unsaturated PNP ester and the reaction product. Competitive acylation of the catalyst **5** by the product and starting material is consistent with the observed product inhibition and partial negative order in product.

**Scheme 3:**
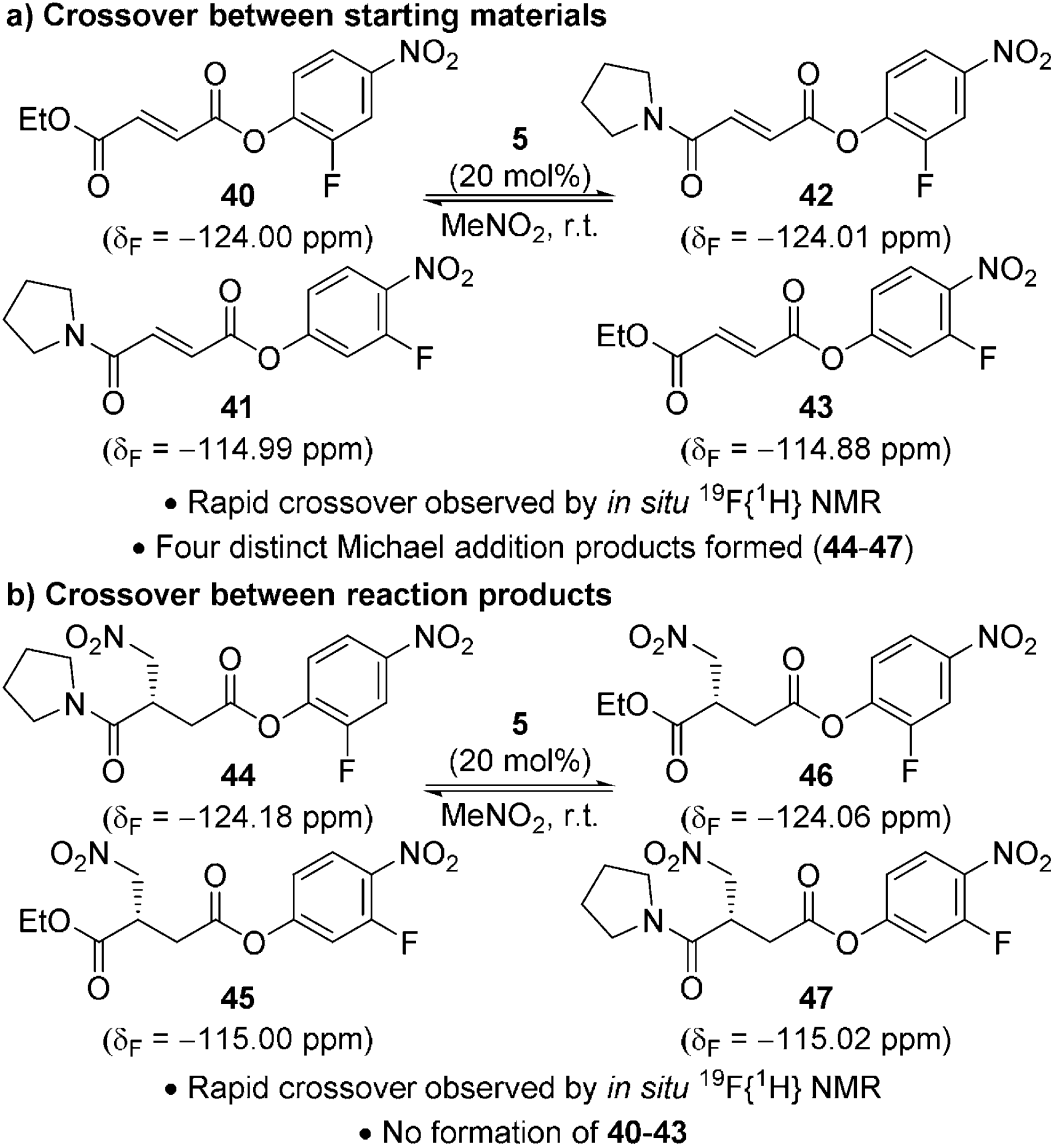
Crossover experiments monitored by in situ ^19^F{^1^H} NMR spectroscopy.

Based on these studies the following catalytic cycle is proposed (Scheme 4). The process begins with rapid and reversible catalyst acylation by the α,β‐unsaturated PNP ester **48** to give α,β‐unsaturated acyl isothiouronium **49**, with the position of equilibrium favoring the free catalyst **5** and α,β‐unsaturated PNP ester **48**. Michael addition of nitronate to α,β‐unsaturated acyl isothiouronium **49**, followed by protonation, gives acyl isothiouronium **51**.^25^ It is conceivable that the *p*‐nitrophenoxide counterion released upon acylation may facilitate deprotonation of nitromethane,^26^ with subsquent protonation of the isothiouronium enolate **50** facilitated by either nitromethane or *p*‐nitrophenol. Finally, catalyst turnover by *p*‐nitrophenoxide gives the Michael addition product **52** and regenerates isothiourea **5**. Based on kinetic studies and the rapid crossover between ^19^F‐labeled α,β‐unsaturated PNP esters **40** and **41** relative to the overall rate of reaction, it is likely that Michael addition of nitronate to α,β‐unsaturated acyl isothiouronium **49** is the turnover rate‐limiting step. Based on previous experimental and computational studies it is believed the α,β‐unsaturated acyl isothiouronium **49** adopts an s‐*cis* conformation, with a *syn*‐coplanar non‐covalent 1,5‐S⋅⋅⋅O interaction between the acyl O and catalyst S providing a conformational lock.7b,7d, 8, 10d, 27 The stereochemical outcome of the process can therefore be rationalized by Michael addition of nitronate to the α,β‐unsaturated acyl isothiouronium **49** *anti*‐ to the stereodirecting phenyl substituent of the isothiourea catalyst.

**Scheme 4:**
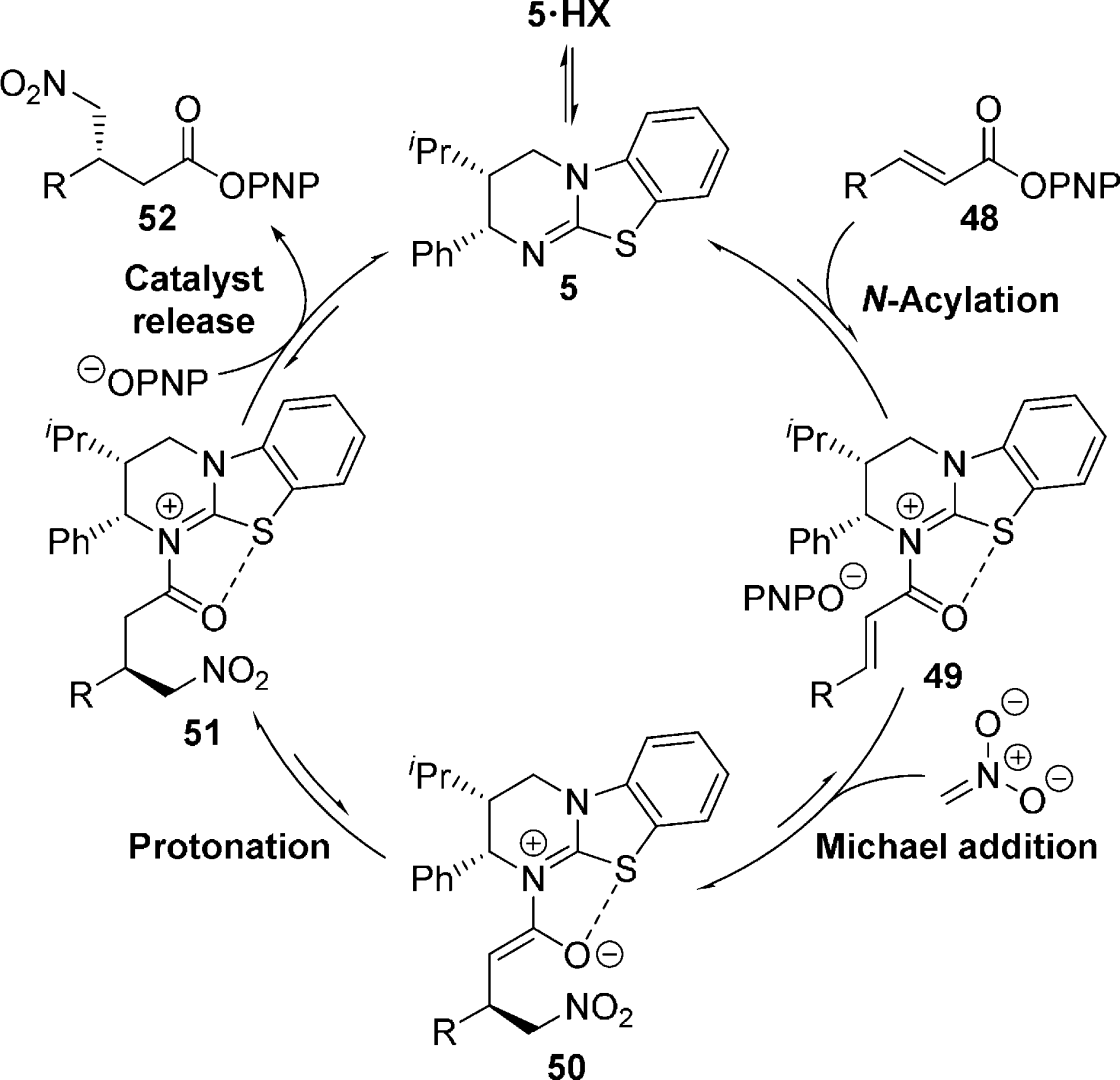
Proposed mechanism.

In conclusion, a new general concept for α,β‐unsaturated acyl ammonium catalysis has been developed which exploits *p*‐nitrophenoxide release from an α,β‐unsaturated *p*‐nitrophenyl ester substrate to facilitate catalyst turnover. This method allows the use of simple nucleophilic reaction partners for the first time. The concept was demonstrated in an enantioselective Michael addition of nitroalkanes to α,β‐unsaturated *p*‐nitrophenyl esters in generally good yield and with excellent enantioselectivity (27 examples, up to 79 % yield, 99:1 er). Mechanistic studies identified rapid and reversible catalyst acylation by the α,β‐unsaturated *p*‐nitrophenyl ester to give a key α,β‐unsaturated acyl isothiouronium intermediate. Product inhibition and catalyst deactivation by protonation were identified under the reaction conditions, and application of a recently‐reported variable time normalization graphical analysis method was required to allow the complex reaction kinetics to be probed. It is hoped that the report of this new reaction paradigm in α,β‐unsaturated acyl ammonium catalysis will enable and encourage further advancement of this burgeoning field.^28^

## Conflict of interest

The authors declare no conflict of interest.
